# Sequential Ni-Pt Decoration on Co(OH)_2_ via Microwave Reduction for Highly Efficient Alkaline Hydrogen Evolution

**DOI:** 10.3390/nano15241876

**Published:** 2025-12-14

**Authors:** Luan Liu, Hongru Liu, Zikang Chen, Genghua Cao, Xiaoyu Wu, Baorui Jia, Xuanhui Qu, Mingli Qin

**Affiliations:** 1 Institute for Advanced Materials and Technology, University of Science and Technology Beijing, Beijing 100083, China; 2 School of Automobile and Transportation, Shenzhen Polytechnic University, Shenzhen 518055, China; 3 Department of Materials Science and Engineering, National University of Singapore, Singapore 117575, Singapore; 4 Shunde Innovation School, University of Science and Technology Beijing, Foshan 301811, China; 5 Beijing Advanced Innovation Center for Materials Genome Engineering, University of Science and Technology Beijing, Beijing 100083, China; 6 Institute of Materials Intelligent Technology, Liaoning Academy of Materials, Shenyang 110167, China

**Keywords:** Ni-Pt@Co(OH)_2_, microwave-assisted synthesis, hydrogen evolution reaction (HER), Pt utilization, interfacial synergy

## Abstract

A rapid solvent-free microwave-assisted strategy was developed to fabricate Pt- and Ni-modified Co(OH)_2_ catalysts for alkaline hydrogen evolution. Among them, Ni-Pt@Co(OH)_2_, prepared via sequential Ni-first then Pt loading, exhibited the best performance. Structural analyses confirmed uniform Pt dispersion with dominant Pt(111) facets, while ICP-MS showed reduced Pt usage compared to Pt@Co(OH)_2_. Electrochemical measurements in 1.0 M KOH revealed an overpotential of 71 mV at 10 mA·cm^−2^, comparable to Pt/C, and a mass activity 4–6.5 times higher across 25–75 mV. EIS demonstrated lower charge-transfer resistance, and stability tests showed negligible degradation after 3000 CV cycles and 11 h continuous operation. The outstanding performance arises from enhanced Pt utilization, abundant conductive sites, and strong Ni-Pt interfacial synergy, highlighting Ni-Pt@Co(OH)_2_ as a promising catalyst for efficient alkaline HER with reduced Pt consumption.

## 1. Introduction

The hydrogen evolution reaction (HER) is a key half-reaction in water electrolysis and plays a pivotal role in the development of sustainable hydrogen-based energy systems [1,2,3,4]. Platinum (Pt) is widely recognized as the most efficient HER catalyst due to its near-zero hydrogen adsorption free energy and superior intrinsic activity [5,6]. However, the high cost and scarcity of Pt severely limit its large-scale application [7,8]. Consequently, designing Pt-based catalysts with maximized Pt utilization and enhanced catalytic efficiency has become a pressing challenge [9,10].

Cobalt hydroxide (Co(OH)_2_), with its low cost, abundance, and tunable layered structure, has recently attracted increasing attention as a promising support material for electrocatalysts [11,12,13,14]. Its two-dimensional nanosheet morphology and high surface area provide abundant anchoring sites for active metals [15,16]. Moreover, coupling Co(OH)_2_ with noble metals can not only stabilize metal nanoparticles but also modulate their electronic structure, thereby improving HER performance [17,18]. Despite these advantages, conventional Pt/Co(OH)_2_ composites often suffer from non-uniform Pt distribution, limited exposure of active sites, and relatively poor mass activity [19].

To overcome these limitations, bimetallic modification strategies have been proposed [20,21,22], in which the synergistic effects between Pt and transition metals (such as Ni, Fe, and Co) can significantly boost catalytic activity and durability [23]. Nickel (Ni), in particular, is an attractive partner for Pt because of its excellent conductivity and its ability to regulate the electronic structure of Pt through interfacial interactions [24,25]. Previous studies have shown that incorporating Ni can lower the charge-transfer resistance [26,27,28,29] and promote hydrogen adsorption/desorption kinetics [30]. However, controlling the loading sequence and dispersion of Ni and Pt remains challenging, and its influence on catalytic performance is not yet fully understood.

In this work, we report a facile and rapid solvent-free microwave-assisted strategy to synthesize Pt- and Ni-decorated Co(OH)_2_ catalysts. By tuning the loading sequence of Pt and Ni, we demonstrate that the sequential deposition of Ni followed by Pt yields Ni-Pt@Co(OH)_2_ with outstanding HER performance. Structural characterizations confirm the uniform dispersion of Pt nanoparticles and the predominant exposure of Pt(111) facets, while electrochemical measurements reveal ultralow overpotential, enhanced mass activity, reduced charge-transfer resistance, and excellent long-term stability. The superior performance is attributed to the synergistic effects between Ni and Pt, which maximize Pt utilization and create abundant active interfacial sites. This study provides valuable insights into rational catalyst design for cost-effective and efficient Pt-based electrocatalysts in alkaline HER.

## 2. Experimental Section

### 2.1. Materials

All reagents were of analytical grade and used without further purification. NiCl_2_ (Shanghai Macklin Biochemical Co., Ltd., Shanghai, China), Co(NO_3_)_2_·6H_2_O (Aladdin Biochemical Technology Co., Ltd. (Shanghai, China)), NaH_2_PO_2_·H_2_O (Sinopharm Chemical Reagent Co., Ltd. (Shanghai, China)), K_2_PtCl_4_ (Shanghai Boer Chemical Reagent Co., Ltd., Shanghai, China), KOH (Aladdin Reagent Co., Ltd. (Shanghai, China)), Ammonium hydroxide solution (25–28 wt%, analytical grade, Xilong Scientific Co., Ltd. (Shantou, China)), Nafion solution (Suzhou Senno Technology Co., Ltd., Suzhou, China), and Pt/C (20 wt% Pt, Beijing Inoke Technology Co., Ltd., Beijing, China) were purchased and used as received.

### 2.2. Synthesis of Noble Metal-Decorated Co(OH)_2_ Electrocatalysts

#### 2.2.1. Synthesis of Porous Co(OH)_2_ Precursors

Cobalt hydroxide precursors were prepared using a water-bath method. Specifically, 0.25 g of cobalt(II) nitrate hexahydrate was dissolved in 50 mL of deionized water, and the container was sealed with plastic wrap perforated with small holes. The solution was magnetically stirred at 600 rpm in an 80 °C water bath. Once the temperature stabilized, 8 mL of ammonia solution (25–28 wt%, analytical grade) was quickly added, and the container was resealed. The solution color gradually changed from dark red to blue and finally to pink, at which point the reaction was stopped and the mixture was allowed to settle. The resulting precipitate was collected, washed several times with anhydrous ethanol by centrifugation, and vacuum-dried at 60 °C for 12 h to obtain porous cobalt hydroxide precursor powder.

#### 2.2.2. Synthesis of M@Co(OH)_2_ (M = Ni, Pt, Etc.) Noble Metal Electrocatalysts

The M@Co(OH)_2_ catalysts were synthesized via a rapid microwave-assisted reduction method, requiring only 1 min. Specifically, 30 mg of Co(OH)_2_ powder, 5 mg of anhydrous NiCl_2_ powder (or 5 mg of K_2_PtCl_4_ powder, or no metal precursor), and 5 mg of NaH_2_PO_2_ were thoroughly ground in a mortar. The homogeneous mixture was then transferred into a 5 mL quartz vial, wetted with deionized water, and subjected to microwave irradiation at 700 W for 1 min. The resulting solid, which formed slight agglomerates after microwave irradiation, was simply ground into a fine powder without any washing or filtration steps and used directly for subsequent experiments, all other catalyst samples were prepared using the same microwave-assisted procedure described above.

#### 2.2.3. Synthesis of 2M@Co(OH)_2_ (M = Ni, Pt) Noble Metal Catalysts

In this work, three types of noble metal catalysts were prepared: (i) Pt and Ni atoms co-loaded on the cobalt hydroxide precursor; (ii) Pt atoms loaded first, followed by Ni atoms; and (iii) Ni atoms loaded first, followed by Pt atoms.

(1) Synthesis of PtNi Co-Decorated Co(OH)_2_ Catalysts

The PtNi@Co(OH)_2_ catalyst was synthesized via a one-step microwave-assisted reduction method. Specifically, 30 mg of Co(OH)_2_ precursor, 10 mg of NaH_2_PO_2_, 5 mg of K_2_PtCl_4_, and 5 mg of NiCl_2_ powders were thoroughly ground, transferred into a 5 mL quartz vial, and wetted with deionized water. The mixture was then subjected to microwave irradiation at 700 W for 1 min. The resulting product was collected after grinding and designated as the PtNi@Co(OH)_2_ catalyst.

(2) Synthesis of Pt-Ni@Co(OH)_2_ Cobalt Hydroxide Catalysts with Sequential Pt-then-Ni Loading

The Pt-Ni@Co(OH)_2_ catalyst was prepared using a two-step microwave-assisted reduction method. First, 30 mg of Co(OH)_2_ precursor, 5 mg of NaH_2_PO_2_, and 5 mg of K_2_PtCl_4_ powders were thoroughly ground, transferred into a 5 mL quartz vial, wetted with deionized water, and subjected to microwave irradiation at 700 W for 1 min to obtain the Pt-Co(OH)_2_ catalyst. Subsequently, the obtained product was mixed and ground with 5 mg of NaH_2_PO_2_ and 5 mg of NiCl_2_ powders, followed by a second microwave treatment (700 W, 1 min). The final product was collected after grinding and designated as the Pt-Ni@Co(OH)_2_ catalyst.

(3) Synthesis of Ni-Pt@Co(OH)_2_ Cobalt Hydroxide Catalysts with Sequential Ni-then-Pt Loading

The Ni-Pt@Co(OH)_2_ catalyst was synthesized via a two-step solvent-free microwave-assisted reduction method. First, 30 mg of Co(OH)_2_ precursor, 5 mg of NaH_2_PO_2_, and 5 mg of NiCl_2_ were thoroughly ground, transferred into a 5 mL quartz vial, wetted with deionized water, and subjected to microwave irradiation at 700 W for 1 min to obtain the Ni-Co(OH)_2_ precursor. Subsequently, the obtained product was mixed and ground with 5 mg of NaH_2_PO_2_ and 5 mg of K_2_PtCl_4_, followed by a second microwave treatment (700 W, 1 min). The final product was collected after grinding and designated as the Ni-Pt@Co(OH)_2_ catalyst.

### 2.3. Characterization

The phase composition of the products was identified by X-ray diffraction (XRD, Rigaku D/max-RB12, Rigaku Corporation, Tokyo, Japan) using Cu Kα radiation in the 2θ range of 10–80° at room temperature. The morphology of the samples was examined by field-emission scanning electron microscopy (FESEM, NOVA NANOSEM 450 FEI Company, Hillsboro, OR, USA). TEM images were obtained using transmission electron microscope (JEM-2010, JEOL Ltd., Tokyo, Japan) operated at an accelerating voltage of 300 kV. For elemental analysis, inductively coupled plasma mass spectrometry (ICP-MS) was performed on an Inductively coupled plasma optical emission spectrometer (ICP-OES 730, Agilent Technologies, Santa Clara, CA, USA) with argon as the carrier gas.

### 2.4. Electrochemical Performance Characterization

Electrochemical measurements were performed in 1.0 M KOH using a standard three-electrode setup, with a Hg/HgO electrode as the reference electrode, a graphite rod as the counter electrode, and a glassy carbon electrode (GCE, 3 mm in diameter) as the working electrode. For the working electrode preparation, 10 mg of catalyst and 2 mg of carbon black were dispersed in a solution containing 20 μL of 5 wt% Nafion, 480 μL of deionized water, and 480 μL of ethanol, followed by ultrasonication to obtain a uniform ink. The GCE was sequentially polished with alumina powders (0.5 μm, 0.3 μm, and 50 nm), rinsed with ethanol, and dried. Subsequently, 2 × 2 μL of the catalyst ink was drop-cast onto the GCE surface and dried for use. Linear sweep voltammetry (LSV) was carried out in 1.0 M KOH at a scan rate of 5 mV·s^−1^ over the potential range of −0.80 to −1.60 V. For chronoamperometric stability tests, 50 μL of catalyst ink was uniformly coated onto a 1 × 1 cm^2^ piece of carbon paper, which served as the working electrode, and i-t measurements were conducted at −1 V in 1.0 M KOH. All electrochemical tests were conducted on a CHI 660E electrochemical workstation (CH Instruments, Shanghai, China). Potentials were calibrated to the reversible hydrogen electrode (RHE) according to the following equation:

E_RHE_ = E_SCE_ + 0.242 V + 0.059 × pH

The geometric current density (j) was normalized to the geometric area of the GCE and used to construct the Tafel plots. The mass activity (mA·mg^−1^_Pt_) was normalized to the mass of Pt in the catalyst. Electrochemical impedance spectroscopy (EIS) was performed in 1.0 M KOH at −0.6 V with an AC perturbation amplitude of 0.01 mV over the frequency range from 100 kHz to 0.01 Hz. The resulting Nyquist plots were fitted using a simplified Randles equivalent circuit, where R_s_ represents the solution and external resistance, and R_ct_ corresponds to the interfacial charge-transfer resistance.

## 3. Results and Discussion

**Figure 1:**
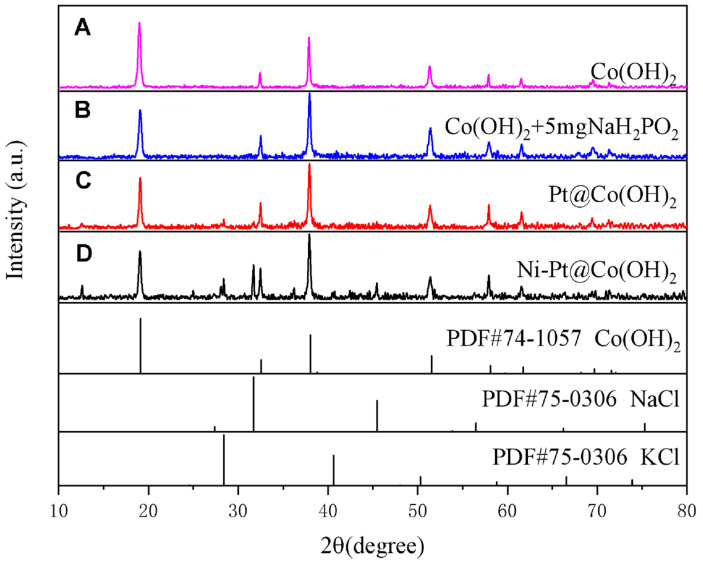
XRD patterns of Co(OH)_2_, Co(OH)_2_ + 5 mg NaH_2_PO_2_, Pt@Co(OH)_2_, and Ni-Pt@Co(OH)_2_.

As shown in Figure 1A, the diffraction peaks at 19.11°, 38.03°, and 51.51° can be indexed to the (001), (011), and (012) planes of Co(OH)_2_ (JCPDS 74-1057), respectively. After the addition of 5 mg NaH_2_PO_2_ followed by microwave reduction (Figure 1B), the XRD pattern remains nearly identical to that of pristine Co(OH)_2_ (Figure 1A), with no additional diffraction peaks observed, indicating that the crystal structure of Co(OH)_2_ is preserved after microwave treatment. When Pt was introduced onto the Co(OH)_2_ precursor (Figure 1C), no diffraction peaks associated with Pt-related were detected in the updated XRD patterns. This is consistent with the relatively low Pt content (~1.21 at.%) and the ultrasmall particle size (as shown in Figure S1), which make the Pt phase difficult to resolve by XRD. ICP-MS quantification (Table 1) shows that the Pt loading in Pt@Co(OH)_2_ is 5.74 wt%. In the XRD pattern of Ni-Pt@Co(OH)_2_ obtained via the two-step microwave assisted reduction (Figure 1D), no diffraction peaks corresponding to Pt or Ni-related were observed. This can be attributed to the relatively low metal loadings and the possibility that Pt and Ni exist in a highly dispersed and poorly crystalline state. Apart from the characteristic reflections of Co(OH)_2_, several peaks originating from residual KCl and NaCl-formed from the chloride-containing precursors during microwave reduction-were also detected. The preserved diffraction profile of Co(OH)_2_ indicates that the two-step microwave treatment does not alter its long-range crystallinity. Although Pt and Ni reflections are absent in the XRD patterns, compositional analyses confirm their successful incorporation. ICP-MS shows that Ni-Pt@Co(OH)_2_ contains 5.34 wt% Pt (Table 1).

**Figure 2:**
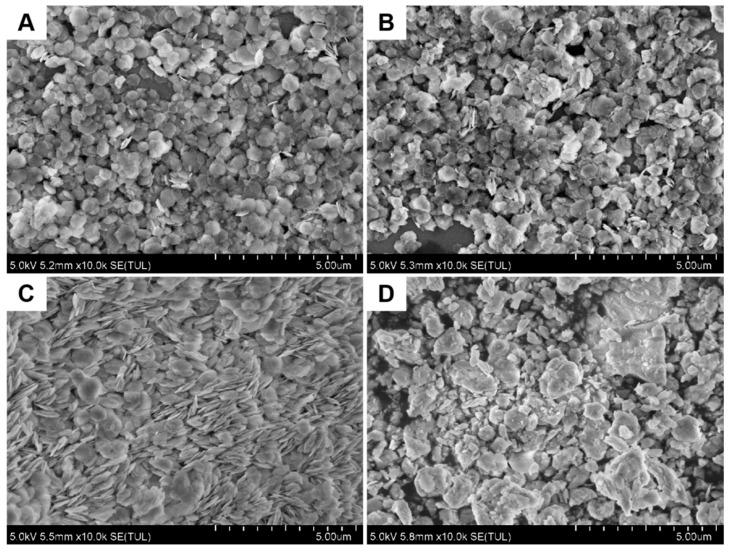
SEM images of (**A**) Co(OH)_2_, (**B**) Co(OH)_2_ + 5 mg NaH_2_PO_2_, (**C**) Pt@Co(OH)_2_, and (**D**) Ni-Pt@Co(OH)_2_ catalysts.

The surface morphologies of Co(OH)_2_, Co(OH)_2_ + 5 mg NaH_2_PO_2_, Pt@Co(OH)_2_, and Ni-Pt@Co(OH)_2_ catalysts were analyzed, and their microstructures are shown in Figure 2. As illustrated in Figure 2A, Co(OH)_2_ exhibits a uniformly dispersed hexagonal sheet-like structure on the silicon substrate. After the first microwave treatment (Figure 2B,C), slight aggregation is observed, whereas a second microwave treatment (Figure 2D) results in more pronounced agglomeration. These results indicate that the introduction of Ni alters the growth behavior of Pt, thereby facilitating the formation of a larger specific surface area. For completeness, SEM images of all comparison samples (Co(OH)_2_, Co(OH)_2_ + 5 mg NaH_2_PO_2_, Pt@Co(OH)_2_, Ni@Co(OH)_2_, PtNi@Co(OH)_2_, Pt-Ni@Co(OH)_2_, and Ni-Pt@Co(OH)_2_) are provided in the Supplementary Information (Figures S2–S8) to confirm the successful preparation of each material.

**Figure 3:**
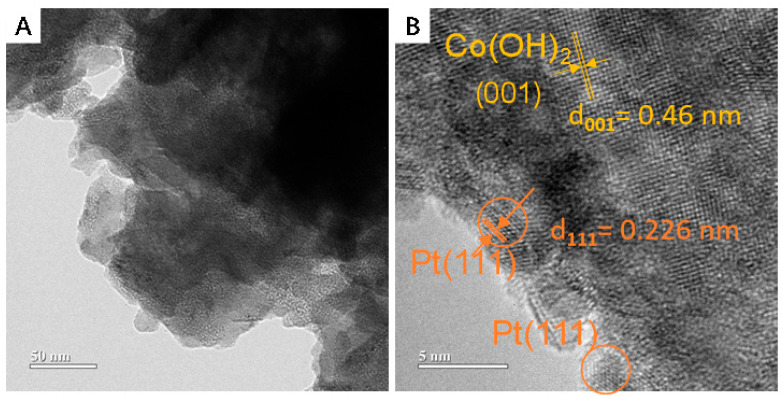
TEM images of Ni-Pt@Co(OH)_2_ at different magnifications (**A**) Low-magnification TEM image, (**B**) High-resolution TEM (HRTEM) image.

The surface microstructure of Ni-Pt@Co(OH)_2_ nanocrystals was further investigated by TEM. As shown in Figure 3A, Co(OH)_2_ exhibits a porous architecture, providing abundant potential active sites for metal loading. The high-resolution TEM image (Figure 3B) reveals that Pt nanoparticles are anchored at Ni-modified regions across the Co(OH)_2_ nanosheet, confirming the successful synthesis of Ni-Pt@Co(OH)_2_. These edge sites are beneficial for enhancing catalytic activity. Moreover, the catalyst surface is predominantly exposed with Pt(111) facets, which possess higher thermodynamic stability and are enriched with step and defect sites, thereby offering additional active centers and further boosting the catalytic performance.

**Figure 4:**
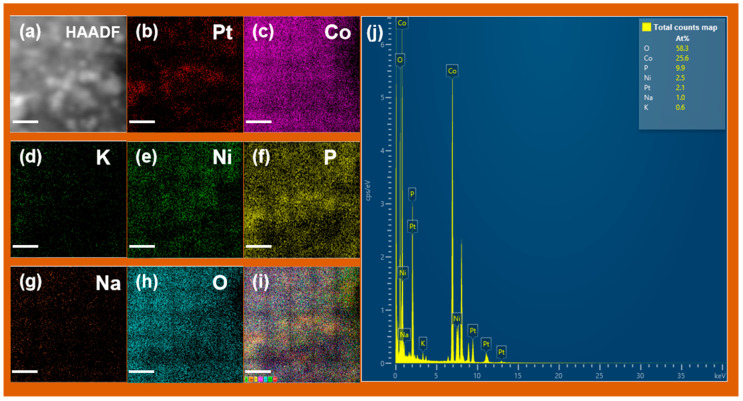
EDS element maps of Ni-Pt@Co(OH)_2_ showing distributions of (**a**) HAADF-STEM image, (**b**) Pt, (**c**) Co, (**d**) K, (**e**) Ni, (**f**) P, (**g**) Na, (**h**) O, (**i**) composite map and (**j**) EDS total counts spectrum with quantitative atomic percentages. The scale bars in (**a**–**i**) correspond to 10 nm.

To further confirm the elemental composition and the spatial distribution of each component, EDS elemental mapping and quantitative EDS analysis were performed on the Ni-Pt@Co(OH)_2_ catalyst (Figure 4). The mapping results show that O and Co are uniformly distributed across the nanosheet framework, while Pt appears as discrete bright spots, consistent with the highly dispersed Pt nanoparticles observed in the TEM images. Quantitative EDS analysis (Figure 4j) further reveals Pt and Ni atomic contents of 2.1 at% and 2.5 at%, respectively. Owing to the O/Co-rich matrix and the surface-sensitive nature of EDS, the Pt atomic percentage obtained from EDS is in good agreement with the Pt loading determined by ICP-MS (~5.34 wt%), while remaining below the detection limit of XRD, consistent with the absence of detectable metal diffraction peaks. The actual Pt content in Ni-Pt@Co(OH)_2_ and Pt@Co(OH)_2_ catalysts was determined, and the results are summarized in Table 1. Compared with Pt@Co(OH)_2_, Ni-Pt@Co(OH)_2_ contains a lower Pt loading yet exhibits superior catalytic activity and stability. When benchmarked against the commercial 20% Pt/C catalyst, Ni-Pt@Co(OH)_2_ demonstrates slightly higher activity while requiring less Pt.

**Figure 5:**
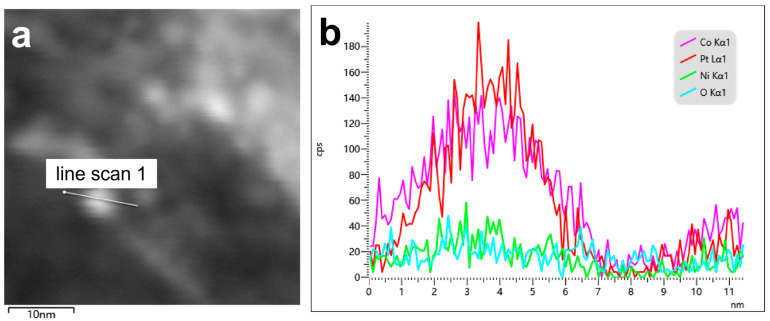
(**a**) STEM image of Ni-Pt@Co(OH)_2_ showing the selected region for elemental line-scan analysis (line scan 1). (**b**) Corresponding EDS line-scan profiles for Co Kα1, Pt Lα1, Ni Kα1, and O Kα1 along the marked line.

To further verify the spatial distribution of Pt and Ni and to directly demonstrate the formation of Ni-Pt interfacial sites, we carried out EDS line-scan analysis on Ni-Pt@Co(OH)_2_ (Figure 5). As shown in Figure 5b, the Co and O signals remain constant across the scanned region, consistent with the uniform Co(OH)_2_ nanosheet framework. In contrast, both Pt and Ni exhibit synchronized and overlapping signal fluctuations along the line profile, clearly indicating that Pt is deposited preferentially on Ni-modified sites rather than randomly distributed. This overlap provides direct evidence for the formation of abundant Ni-Pt interfacial domains. The line-scan analysis in Figures S9 and S10 further confirms this behavior across multiple regions of the nanosheet, demonstrating excellent reproducibility of the Ni-Pt interfacial distribution. These results, together with the mapping data, unequivocally verify the homogeneous dispersion of Pt, its intimate contact with Ni. These interfacial features can be rationalized by the nucleation behavior of Pt on Ni-rich surfaces. The Ni-first loading strategy generates Ni-rich and more conductive surface regions that provide favorable heterogeneous nucleation sites for Pt. Previous studies have shown that Pt nucleates preferentially on Ni substrates rather than in homogeneous solution, indicating that Ni-rich interfaces effectively lower the nucleation barrier and promote uniform Pt deposition [31,32]. Moreover, Ni incorporation has been reported to modulate Pt lattice strain and thermodynamically stabilize Pt(111)-terminated structures during growth [5]. These literature-supported nucleation effects align well with our observations of highly dispersed Pt nanoparticles and dominant Pt(111) facet exposure in Ni-Pt@Co(OH)_2_, elucidating the structural origin of the superior HER performance. It should be noted that the slight fluctuations in the Co Kα signal along the line-scan originate from local thickness variations in the Co(OH)_2_ nanosheet and beam-scattering enhancement induced by Pt/Ni nanoparticles, rather than from changes in Co composition.

**Figure 6:**
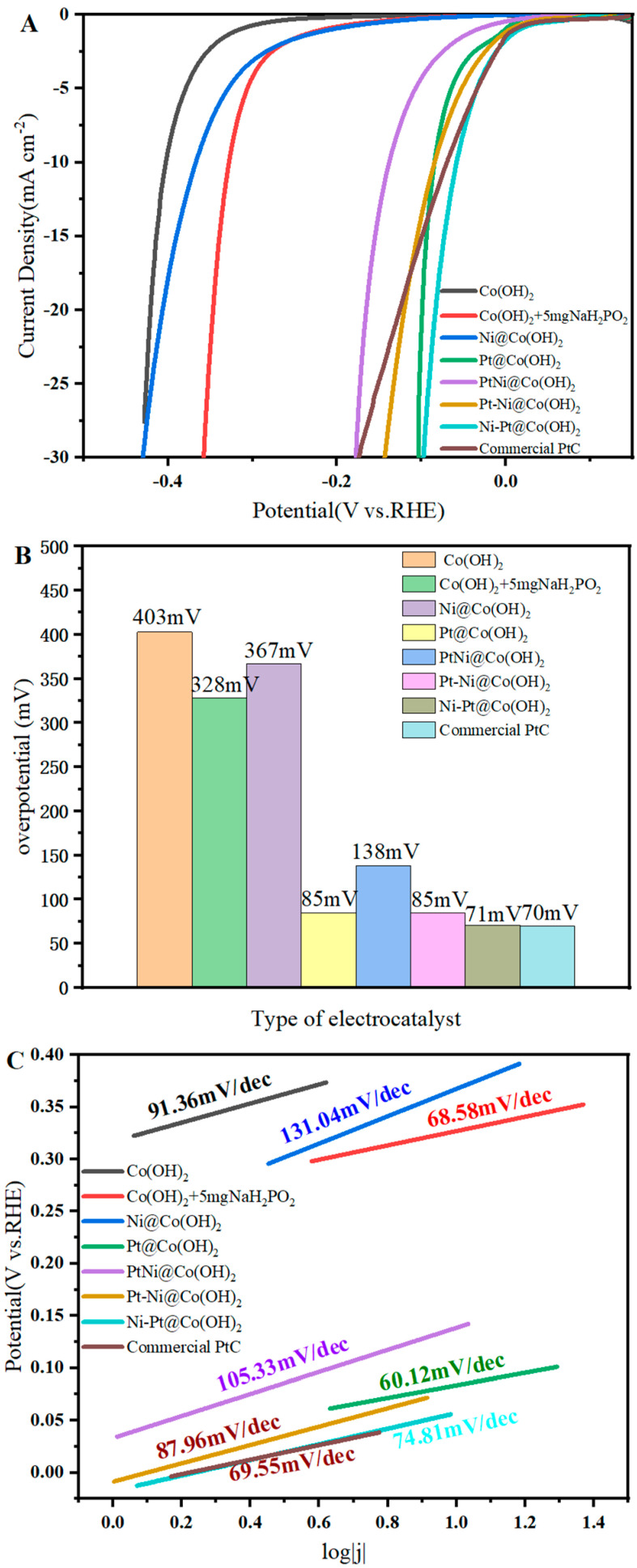
Electrocatalytic activity of different catalysts in 1.0 M KOH: (**A**) LSV curves; (**B**) overpotentials at a geometric current density of 10 mA·cm^−2^; (**C**) Tafel plots.

The catalytic activity of the as-prepared catalysts was evaluated using linear sweep voltammetry (LSV) in a standard three-electrode system with 1.0 M KOH electrolyte, in order to assess the feasibility of Pt and Ni modification on the Co(OH)_2_ precursor. For comparison, Ni-Pt@Co(OH)_2_ and Pt@Co(OH)_2_ were benchmarked against the commercial Pt/C catalyst. As shown in Figure 6A, the LSV curves of Co(OH)_2_, Co(OH)_2_ + 5 mg NaH_2_PO_2_, Ni@Co(OH)_2_, Pt@Co(OH)_2_, PtNi@Co(OH)_2_, Ni-Pt@Co(OH)_2_, Pt-Ni@Co(OH)_2_, and commercial Pt/C were recorded in 1.0 M KOH. Among these, Ni-Pt@Co(OH)_2_ exhibits the best catalytic activity. The bar chart in Figure 6B shows that its overpotential at 10 mA·cm^−2^ is 71 mV, which is nearly identical to that of commercial Pt/C (70 mV). Figure 6C further compares the Tafel slopes of the corresponding catalysts.

**Figure 7:**
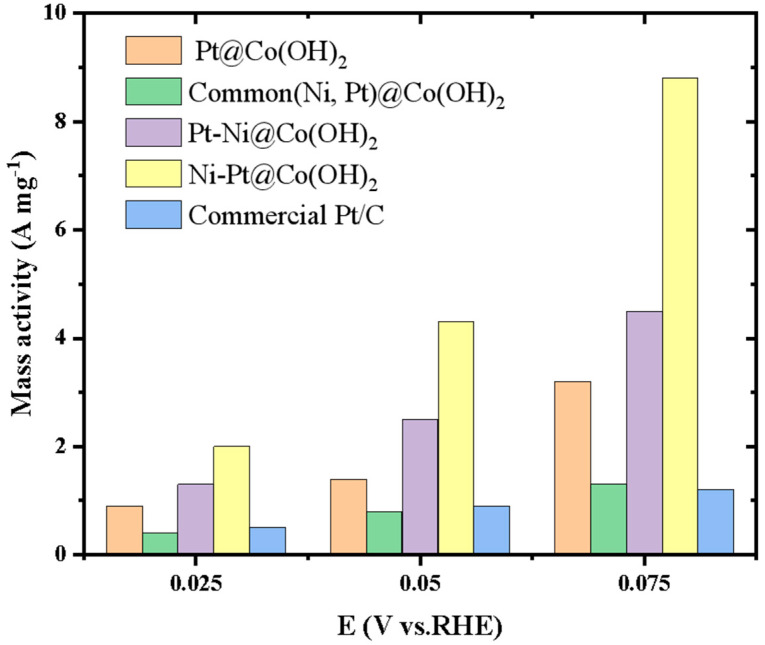
Bar chart comparing the Pt mass activities of different Pt-based catalysts at various overpotentials in 1.0 M KOH.

Figure 7 compares the mass current densities of different noble-metal catalysts (Pt@Co(OH)_2_, PtNi@Co(OH)_2_, Pt-Ni@Co(OH)_2_, Ni-Pt@Co(OH)_2_) and commercial Pt/C in alkaline electrolyte. The results clearly show that Ni-Pt@Co(OH)_2_ exhibits the highest mass activity at all tested potentials: 2.01 A·mg^−1^ at 25 mV, which is four times that of Pt/C (0.48 A·mg^−1^); 4.29 A·mg^−1^ at 50 mV, 4.9 times higher than Pt/C (0.87 A·mg^−1^); and 8.64 A·mg^−1^ at 75 mV, 6.5 times higher than Pt/C (1.32 A·mg^−1^). This superior performance can be attributed to the sequential loading strategy, in which Ni is first deposited on the nanosheet Co(OH)_2_ substrate, followed by Pt. This loading sequence facilitates a more homogeneous distribution of Pt at Ni-Pt interfacial regions, thereby increasing conductive sites and forming Pt-Ni interfacial layers. The abundant exposure of Pt atoms significantly enhances Pt utilization, while the complementary roles of Ni and Pt create strong synergistic effects, leading to markedly improved catalytic activity.

**Figure 8:**
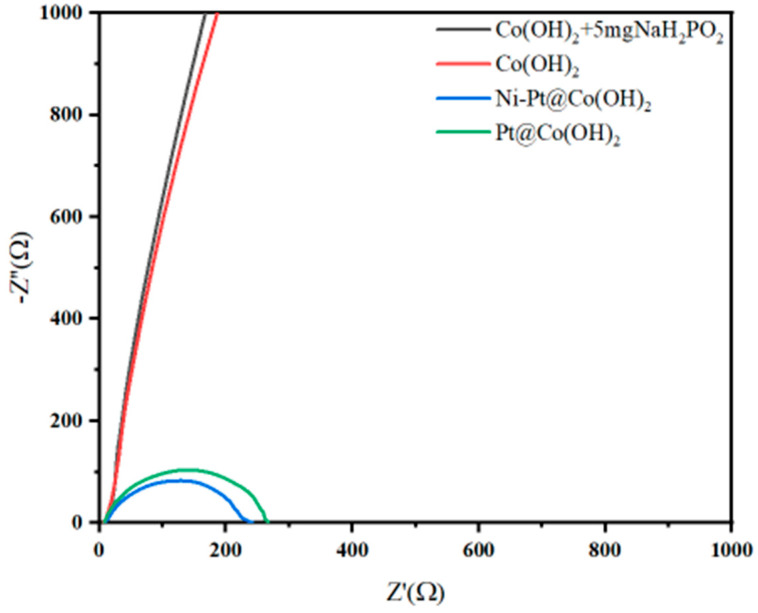
EIS Nyquist plots of different catalysts.

Ni-Pt@Co(OH)_2_ and Pt@Co(OH)_2_ exhibit superior electron transport properties and lower interfacial resistance, as evidenced in the EIS measurements by their smaller semicircle diameters, corresponding to lower charge-transfer resistance (Rct). This indicates a more efficient electron transfer process at the electrode/electrolyte interface, which facilitates the electrochemical reaction. As shown in Figure 8, the Rct values of Ni-Pt@Co(OH)_2_ and Pt@Co(OH)_2_ are significantly lower than those of Co(OH)_2_ and Co(OH)_2_ + 5 mg NaH_2_PO_2_, with Ni-Pt@Co(OH)_2_ displaying the lowest Rct, consistent with its outstanding catalytic activity.

To gain further insight into the role of the loading sequence, the HER activity of PtNi@Co(OH)_2_, Pt-Ni@Co(OH)_2_, and Ni-Pt@Co(OH)_2_ was comparatively analyzed. The co-loading route (PtNi@Co(OH)_2_) leads to the simultaneous nucleation of Pt and Ni, resulting in a less controlled distribution of Pt-Ni interfacial sites and therefore only moderate activity. In the Pt-first route (Pt-Ni@Co(OH)_2_), Pt nanoparticles are first deposited onto the Co(OH)_2_ surface and act as anchoring sites for subsequently introduced Ni species. During the second step, Ni preferentially grows on these Pt nuclei and tends to form a partial overlayer, which shields part of the Pt surface. This reduces the density of exposed Pt active sites and leads to inferior HER activity compared with Ni-Pt@Co(OH)_2_. By contrast, pre-loading Ni (Ni-Pt@Co(OH)_2_) produces a more conductive Ni-containing surface environment that provides favorable nucleation sites for Pt. This loading sequence maximizes the exposure of Pt at Pt-Ni interfacial sites, lowers the charge-transfer resistance, and significantly enhances the Pt mass activity.

**Figure 9:**
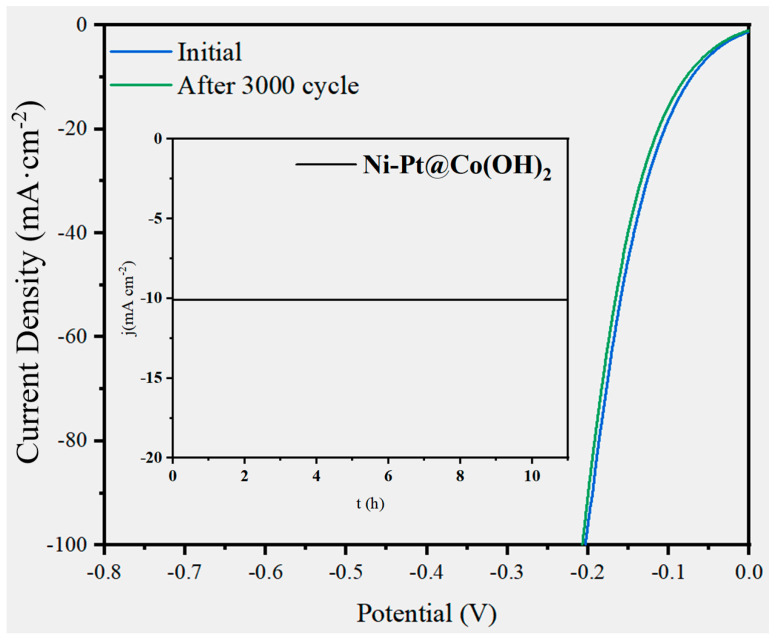
LSV curves of the Ni-Pt@Co(OH)_2_ catalyst before and after 2000 CV cycles, with the inset showing the chronoamperometric i-t curve at a constant potential.

Stability is a critical criterion for evaluating catalyst performance. To this end, cyclic voltammetry (CV) cycling and chronoamperometry (i-t) measurements were conducted to assess the durability of Ni-Pt@Co(OH)_2_. As shown in Figure 9, the polarization curves remain almost unchanged after 3000 CV cycles, indicating excellent cycling stability. In addition, under a constant potential of −1 V in 1.0 M KOH, Ni-Pt@Co(OH)_2_ operated steadily for 11 h without noticeable degradation (inset of Figure 9). The outstanding stability is likely attributed to the strong adhesion and uniform dispersion of Pt quantum dots on the Ni surface, as well as the multiphase synergistic effects at the Ni-Pt interface.

## 4. Conclusions

In summary, a series of Pt- and Ni-modified Co(OH)_2_ catalysts were successfully synthesized via a rapid, solvent-free microwave-assisted reduction strategy. Among them, the sequentially prepared Ni-Pt@Co(OH)_2_ catalyst, obtained by first depositing Ni on Co(OH)_2_ followed by Pt, exhibited the most outstanding hydrogen evolution performance in alkaline electrolyte. Structural characterizations confirmed the uniform dispersion of Pt nanoparticles with predominant exposure of Pt(111) facets, while ICP-MS analysis revealed a reduced Pt loading compared to Pt@Co(OH)_2_. Electrochemical evaluations demonstrated that Ni-Pt@Co(OH)_2_ required an overpotential of only 71 mV at 10 mA·cm^−2^, nearly identical to commercial Pt/C, and delivered mass activities 4–6.5 times higher than Pt/C across different overpotentials. Furthermore, EIS measurements indicated its lowest charge-transfer resistance, and durability tests confirmed remarkable stability after 3000 CV cycles and 11 h of continuous operation. The superior catalytic activity and stability of Ni-Pt@Co(OH)_2_ are attributed to the sequential loading strategy, which promotes uniform Pt dispersion, maximizes Pt utilization, increases conductive sites, and induces strong Ni-Pt interfacial synergy. This study not only provides new insights into the rational design of Pt-based bimetallic catalysts but also offers a simple and efficient approach to developing cost-effective and durable electrocatalysts for alkaline hydrogen evolution.

## Figures and Tables

**Table 1:** Actual elemental contents of the catalysts determined by ICP-MS.

Catalysts	Pt[wt%]1	Pt[wt%]2	Pt[wt%]3	Average Pt Content [wt%]
Pt@Co(OH)_2_	5.6926%	5.9932%	5.5263%	5.7374%
Ni-Pt@Co(OH)_2_	5.6653%	5.2463%	5.1120%	5.3412%

## Data Availability

The original contributions presented in this study are included in the article/Supplementary Material. Further inquiries can be directed to the corresponding authors.

## Supplementary Materials

The following supporting information can be downloaded at: https://www.mdpi.com/article/10.3390/nano15241876/s1.
