# Supplementary material for: Sequential Ni-Pt Decoration on Co(OH)2 via Microwave Reduction for Highly Efficient Alkaline Hydrogen Evolution
Source: Nanomaterials (Basel). 2025 Dec 14;15(24):1876. doi: 10.3390/nano15241876 (PMC12736261; doi:10.3390/nano15241876)
Supplement: Supplementary file 1 [file nanomaterials-15-01876-s001.zip › nanomaterials-3970334-supplementary.pdf]

Supporting Information

Sequential Ni-Pt Decoration on $Co(OH)_2$ via Microwave Reduction for Highly Efficient Alkaline Hydrogen Evolution

Luan Liu [1,*,†], Hongru Liu [1,†], Zikang Chen [1], Genghua Cao [2,*], Xiaoyu Wu [2], Baorui Jia [1,3,4,*], Xuanhui Qu [1,5] and Mingli Qin [1,5,6,*]

[1] Institute for Advanced Materials and Technology, University of Science and Technology Beijing, Beijing 100083, China
[2] School of Automobile and Transportation, Shenzhen Polytechnic University, Shenzhen 518055, China
[3] Department of Materials Science and Engineering, National University of Singapore, Singapore 117575, Singapore
[4] Shunde Innovation School, University of Science and Technology Beijing, Foshan 301811, China
[5] Beijing Advanced Innovation Center for Materials Genome Engineering, University of Science and Technology Beijing, Beijing 100083, China
[6] Institute of Materials Intelligent Technology, Liaoning Academy of Materials, Shenyang 110167, China
* Correspondence: b2230886@ustb.edu.cn (L.L.); caogenghua@szpu.edu.cn (G.C.); jiabaorui@ustb.edu.cn (B.J.); qinml@mater.ustb.edu.cn (M.Q.)
† These authors contributed equally to this work.

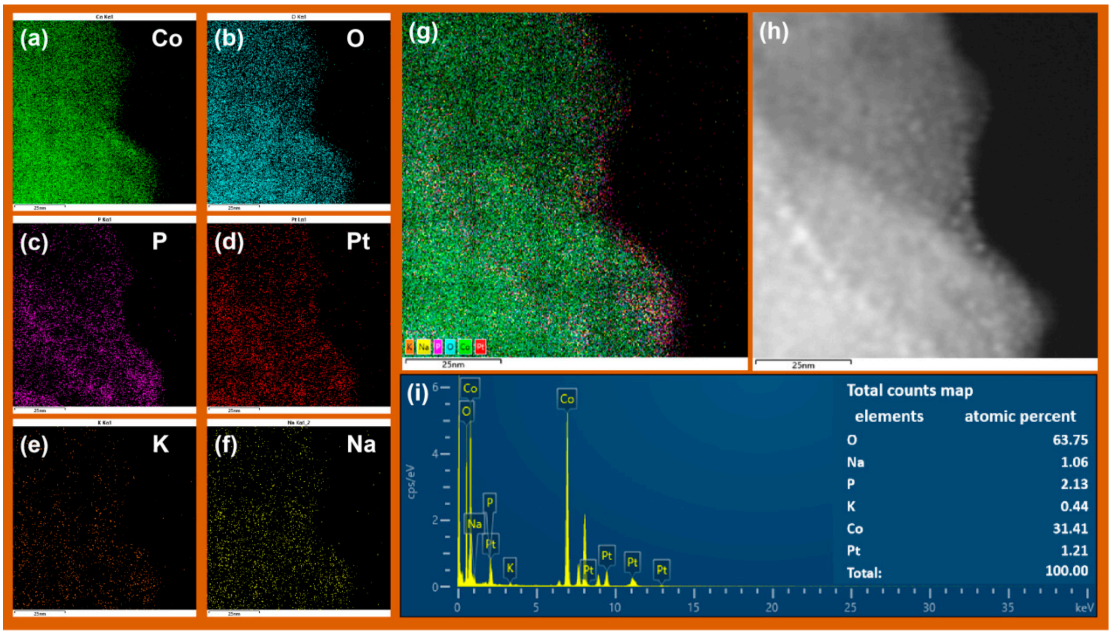

Figure S1. EDS element maps of Pt@Co(OH)$_2$ showing distributions of (a) Co, (b) O, (c) P, (d) Pt, (e) K, (f)Na, (g) composite map and (h) EDS total counts spectrum with quantitative atomic percentages.

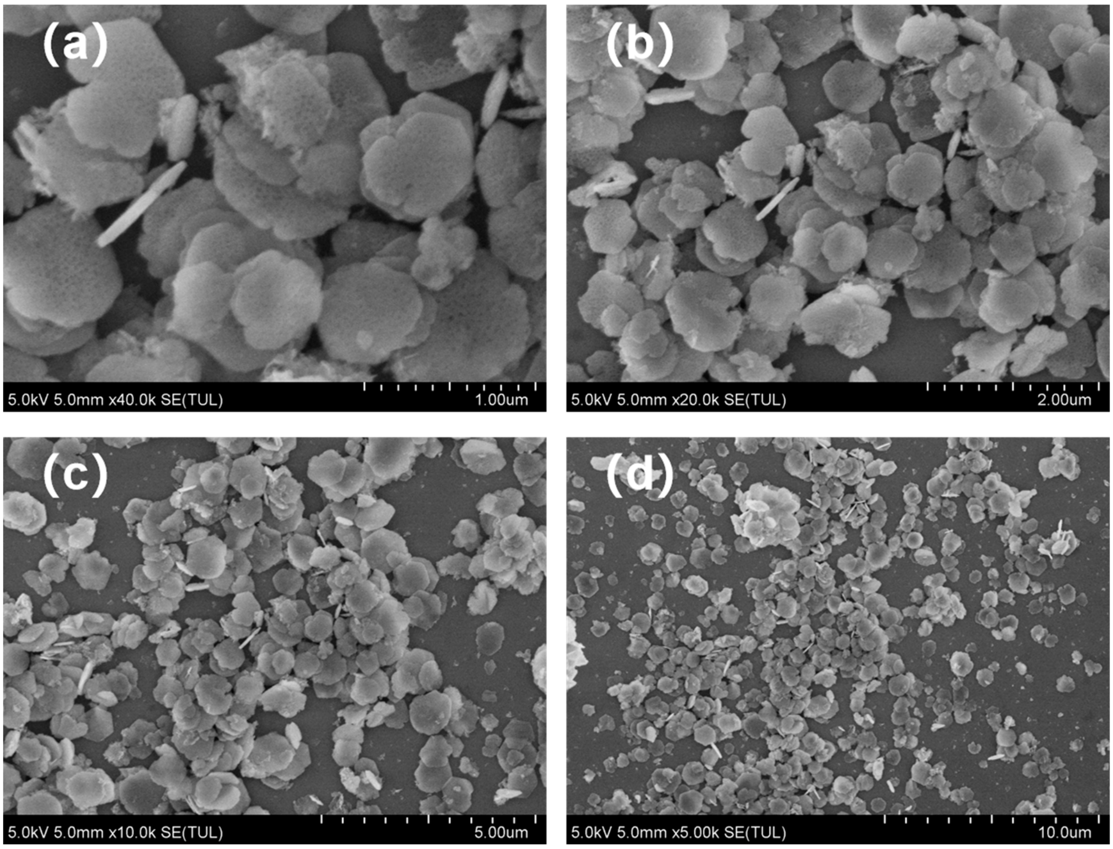

Figure S2. SEM images of $Co(OH)_2$ acquired under different magnifications.

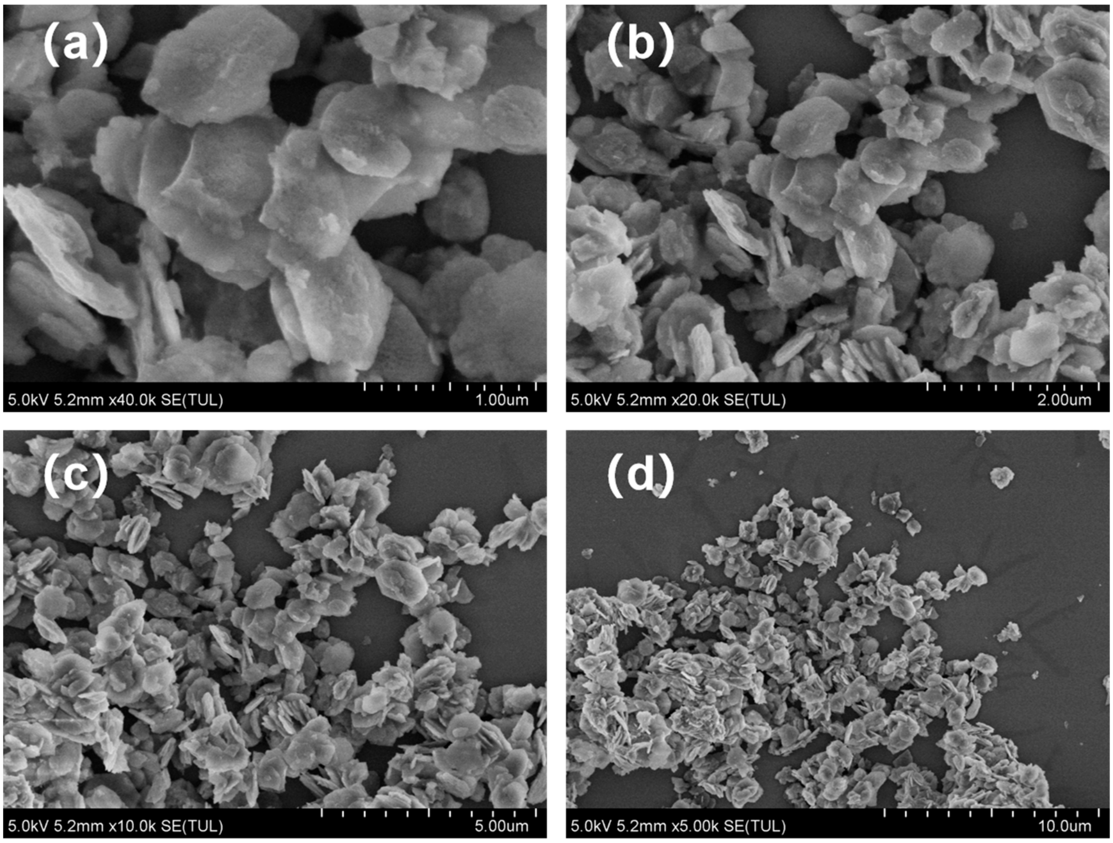

Figure S3. SEM images of $Co(OH)_2$ +5 mg NaH_2PO_2 acquired under different magnifications.

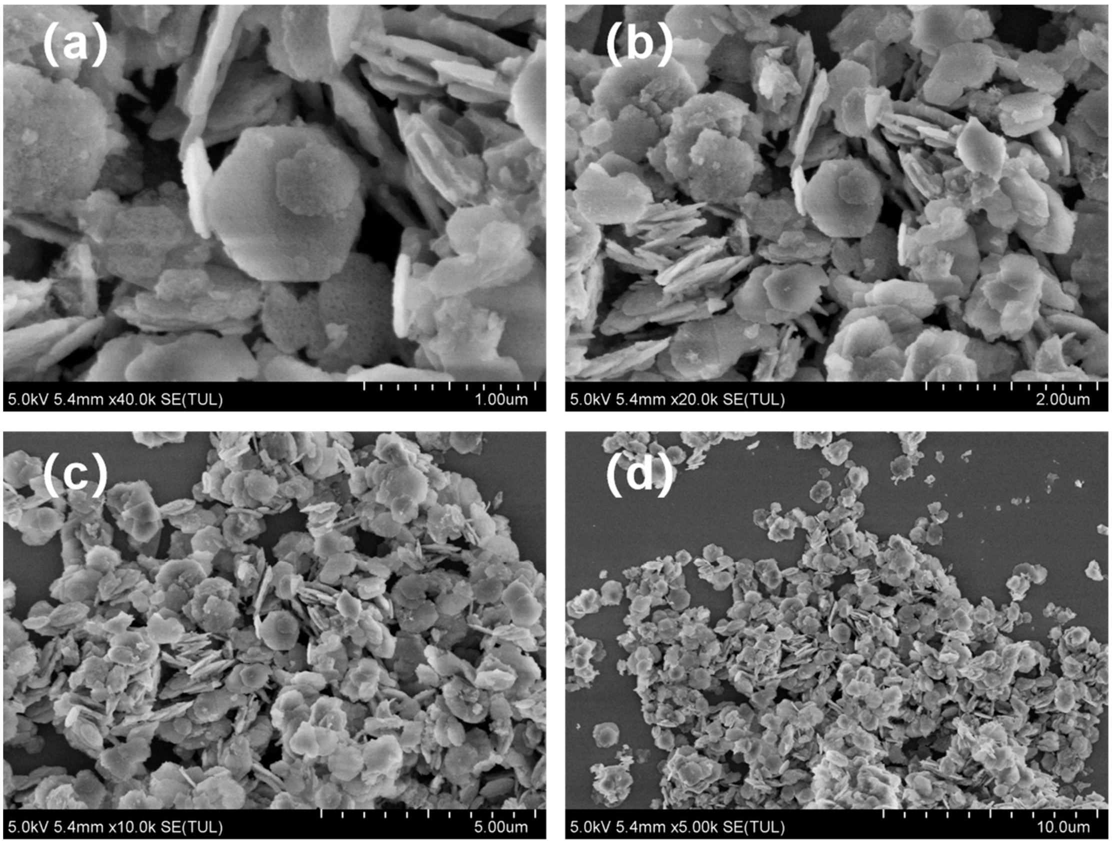

Figure S4. SEM images of Ni@Co(OH)$_2$ acquired under different magnifications.

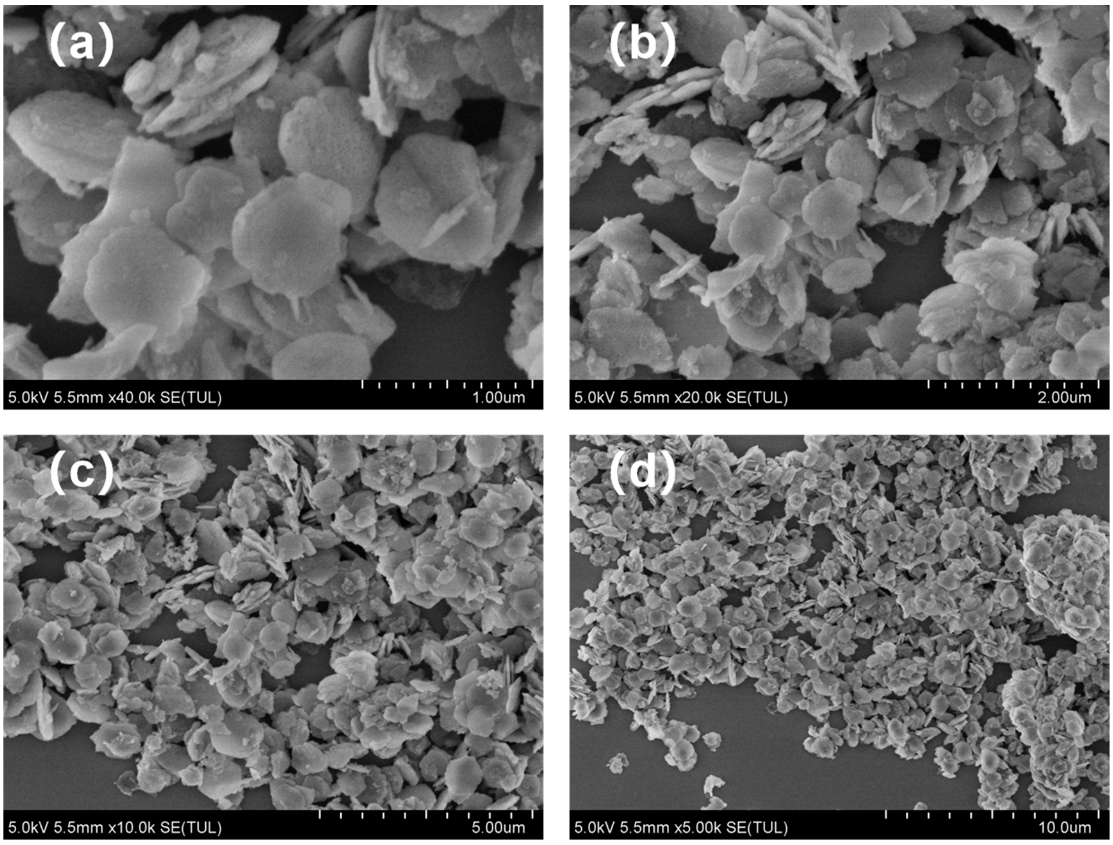

Figure S5. SEM images of Pt@Co(OH)$_2$ acquired under different magnifications.

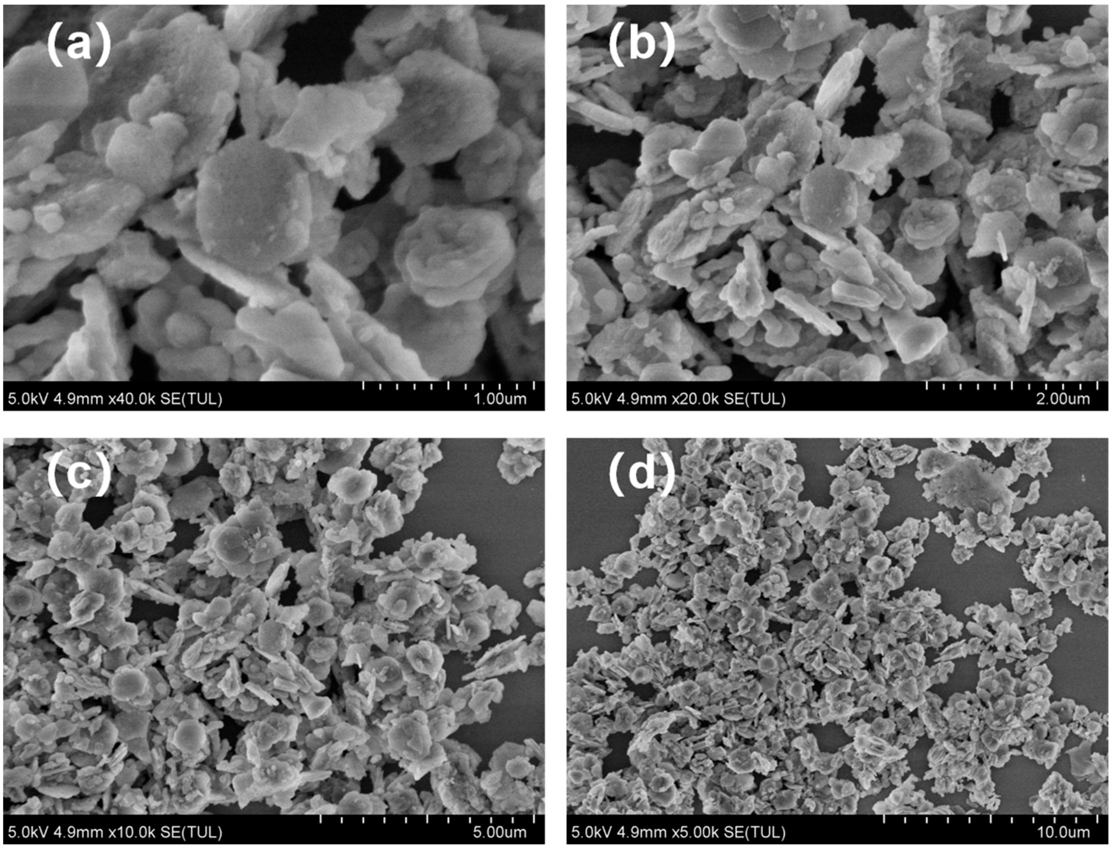

Figure S6. SEM images of PtNi@$Co(OH)_2$ acquired under different magnifications.

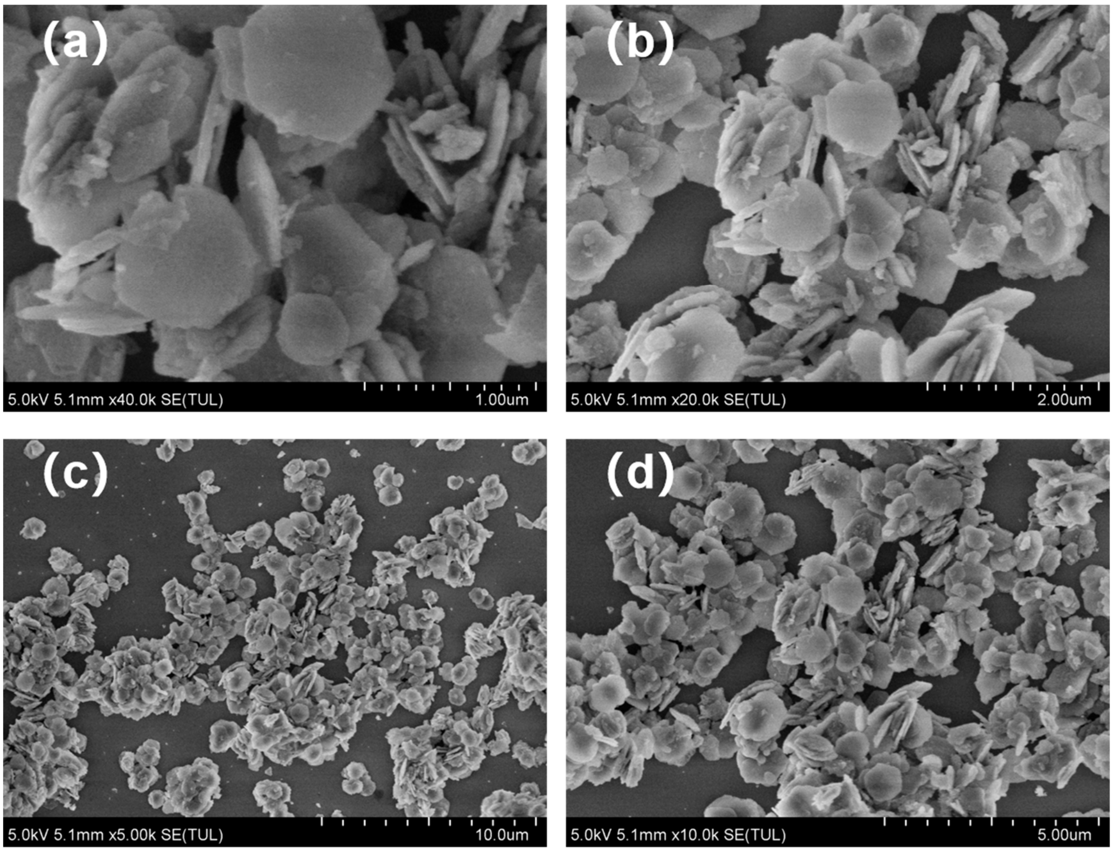

Figure S7. SEM images of Pt-Ni@$Co(OH)_2$ acquired under different magnifications.

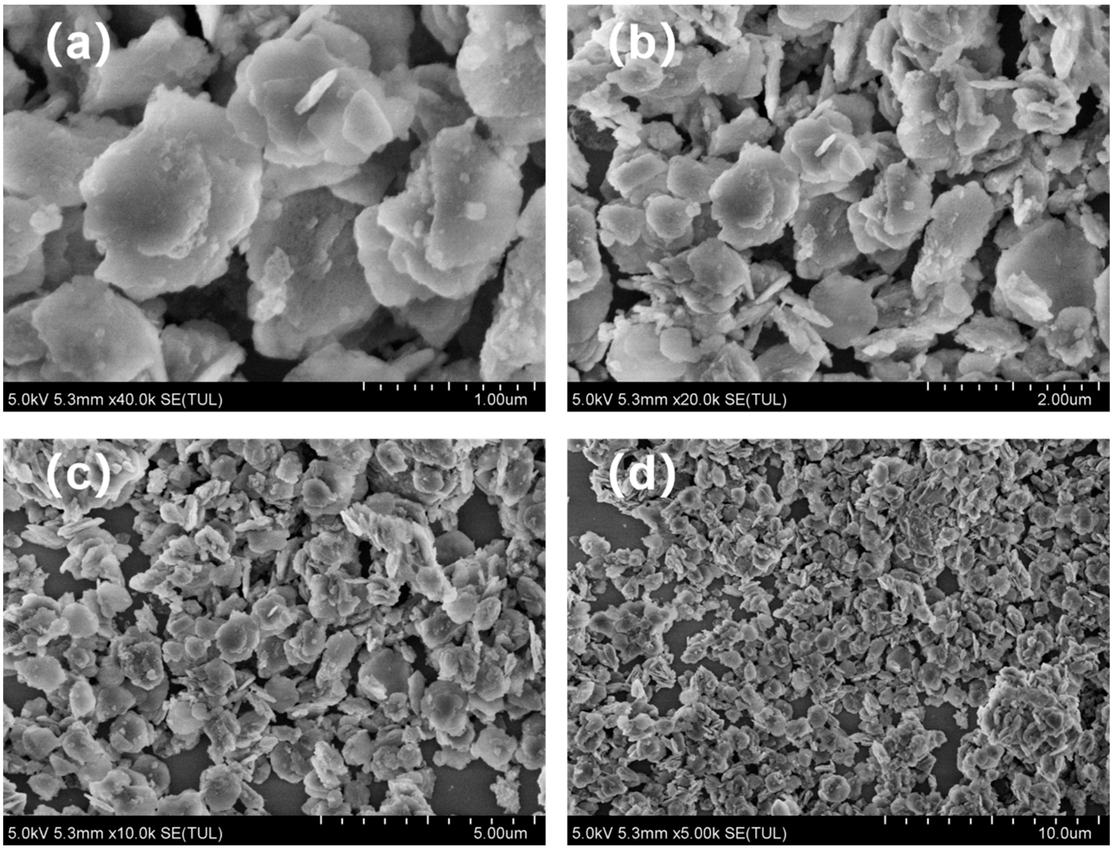

Figure S8. SEM images of Ni-Pt@$Co(OH)_2$ acquired under different magnifications.

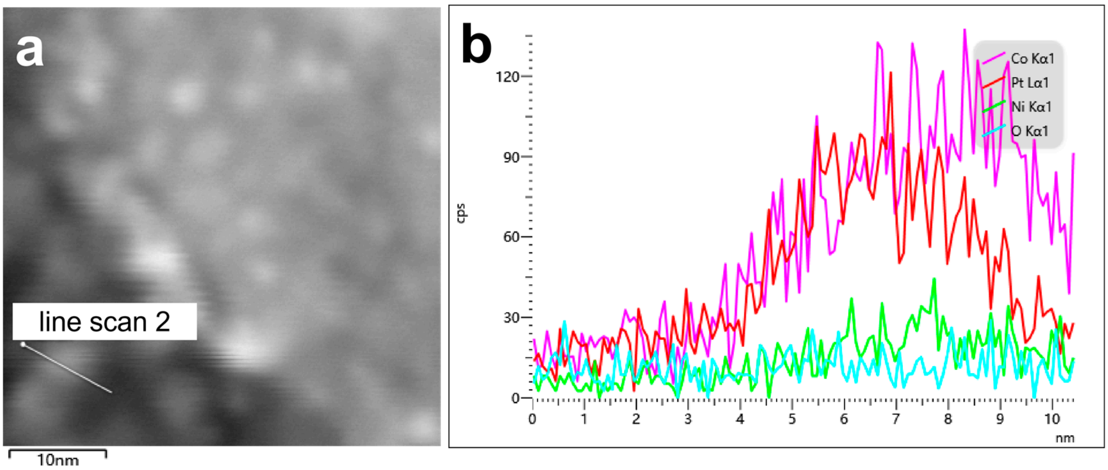

Figure S9. (a) STEM image of Ni-Pt@Co(OH)$_2$ showing the selected region for elemental line-scan analysis (line scan 1). (b) Corresponding EDS line-scan profiles for Co Kα1, Pt Lα1, Ni Kα1, and O Kα1 along the marked line.

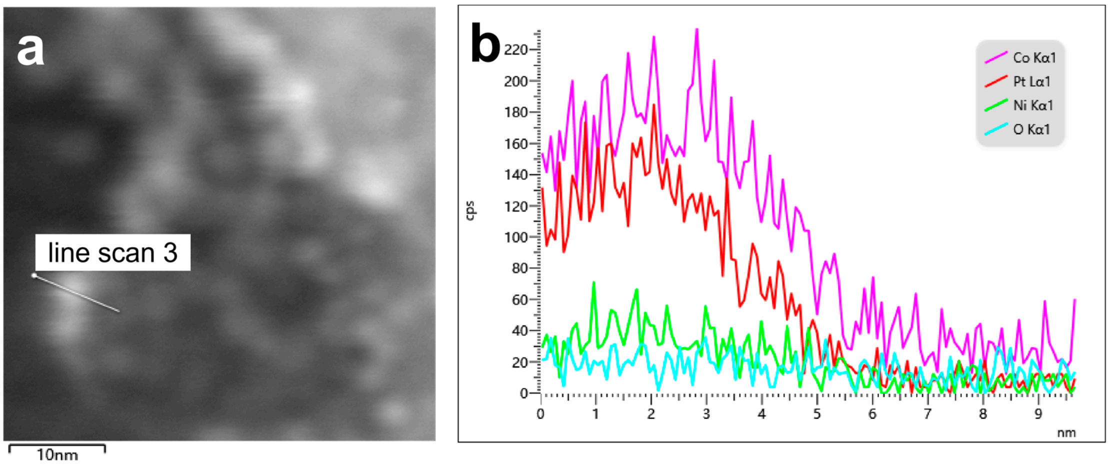

Figure S10. (a) STEM image of Ni-Pt@Co(OH)$_2$ showing the selected region for elemental line-scan analysis (line scan 3). (b) Corresponding EDS line-scan profiles for Co Kα1, Pt Lα1, Ni Kα1, and O Kα1 along the marked line.
